# Subcellular Localization of Iron and Heme Metabolism Related Proteins at Early Stages of Erythrophagocytosis

**DOI:** 10.1371/journal.pone.0042199

**Published:** 2012-07-30

**Authors:** Constance Delaby, Christiane Rondeau, Cécile Pouzet, Alexandra Willemetz, Nathalie Pilard, Michel Desjardins, François Canonne-Hergaux

**Affiliations:** 1 INSERM, U773, Centre de Recherche Bichat Beaujon CRB3, Paris, France; 2 CHU de Montpellier - Hôpital St. Eloi, Institut de Recherches en Biothérapie (IRB), Biochimie - Protéomique Clinique, Monpellier, France; 3 Département de Pathologie et Biologie Cellulaire, Université de Montréal, Montreal, Québec, Canada; 4 Institut Fédératif de Recherche 02, UFR de Médecine Paris, Paris, France; 5 Centre de Recherche de Gif-sur-Yvette, UPR 2301, CNRS, Institut de Chimie des Substances Naturelles (ICSN), Gif-sur-Yvette, France; 6 INSERM U1043-CPTP, Toulouse, France; 7 CNRS, U5282, Toulouse, France; 8 Université de Toulouse, UPS, Centre de Physiopathologie de Toulouse Purpan (CPTP), Toulouse, France; Institut national de la santé et de la recherche médicale (INSERM), France

## Abstract

**Background:**

Senescent red blood cells (RBC) are recognized, phagocytosed and cleared by tissue macrophages. During this erythrophagocytosis (EP), RBC are engulfed and processed in special compartments called erythrophagosomes. We previously described that following EP, heme is rapidly degraded through the catabolic activity of heme oxygenase (HO). Extracted heme iron is then either exported or stored by macrophages. However, the cellular localization of the early steps of heme processing and iron extraction during EP remains to be clearly defined.

**Methodology/Principal Findings:**

We took advantage of our previously described cellular model of EP, using bone marrow-derived macrophages (BMDM). The subcellular localization of both inducible and constitutive isoforms of HO (HO-1 and HO-2), of the divalent metal transporters (Nramp1, Nramp2/DMT1, Fpn), and of the recently identified heme transporter HRG-1, was followed by fluorescence and electron microscopy during the earliest steps of EP. We also looked at some ER [calnexin, glucose-6-phosphatase (G6Pase) activity] and lysosomes (Lamp1) markers during EP. In both quiescent and LPS-activated BMDM, Nramp1 and Lamp1 were shown to be strong markers of the erythrophagolysosomal membrane. HRG-1 was also recruited to the erythrophagosome. Furthermore, we observed calnexin labeling and G6Pase activity at the erythrophagosomal membrane, indicating the contribution of ER in this phagocytosis model. In contrast, Nramp2/DMT1, Fpn, HO-1 and HO-2 were not detected at the membrane of erythrophagosomes.

**Conclusions/Significance:**

Our study highlights the subcellular localization of various heme- and iron-related proteins during early steps of EP, thereby suggesting a model for heme catabolism occurring outside the phagosome, with heme likely being transported into the cytosol through HRG1. The precise function of Nramp1 at the phagosomal membrane in this model remains to be determined.

## Introduction

Macrophages, specialized cells of the immune system, are responsible for the ingestion and elimination of various particles such as pathogens, damaged or apoptotic cells. Senescent red blood cells (RBC) are one of macrophages’ targets and are specifically recognized, engulfed and degraded through the EP pathway [1], [2]. By means of EP, macrophages play key roles in iron metabolism by recycling and storing heme iron from ingested RBC. Heme iron recycling by macrophages represents the largest pathway of iron efflux in the body. However, the cellular and molecular steps of this process remain so far incompletely understood [3], [4]. After internalization of effete erythrocytes into a phagosome defined as an erythrophagosome, heme of hemoglobin is rapidly degraded by the catabolic activity of heme oxygenase (HO), leading to the release of iron, CO and biliverdin. Heme iron reaches the cytoplasmic labile iron pool and is either stored into ferritin or exported through the activity of the cellular iron exporter ferroportin (Fpn) [5], [6]. The HO system consists in two major isoenzymes, known as HO-1 (stress-inducible and ubiquitous) and HO-2 (constitutively expressed, mostly restricted to brain and testis) [7]. HO-1 appears to be the most important isoform for heme catabolism in macrophages [8], [9]. However, the exact localization of heme catabolism inside macrophages remains to be elucidated. One hypothesis depicted in the literature implies the catabolism of heme inside the erythrophagosome. Such a model requires the recruitment of HO-1 to the erythrophagosome or at its membrane with subsequent transport of iron into the cytosol through an iron transporter expressed at the phagosomal membrane. Proteins of the Nramp (Natural resistance-associated macrophage protein) family, Nramp1 [10], [11] or Nramp2/DMT1 (Divalent Metal Transporter1) [12], [13], the first iron transporter identified in mammals, could play such role. On the other hand, heme catabolism could occur outside the phagosomal compartment (into the cytosol) as described in other models. One would therefore expect to detect appropriate heme transporter(s) at the phagosomal membrane. Various heme carrier proteins and transporters have been described [14], [15] and two heme transporters were recently identified in non-polarized cells such as macrophages: HCP1 (Heme carrier protein 1) [16] and HRG1 (Heme responsive gene1) [17]. However the role of heme transporters in EP is ill-defined.

Characterization of the erythrophagosome (nature, composition) is therefore important to shed light on macrophage heme processing and iron recycling pathways. Indeed, the process of phagocytosis by professional cells involves remodeling of the cell surface, cytoskeletal rearrangement and a dynamic succession of membrane fusion/fission of various endocytic compartments leading to formation and maturation of a phagolysosome [18], [19], [20]. During this process, phagosomes sequentially fuse with early endosomes, late endosomes and lysosomes, thus allowing the acquirement of new proteins and hence new functions to maturing phagosomes. The ER has also been proposed to contribute to the phagosomal membrane formation [21], [22], [23]. Importantly, the maturation of phagosome in macrophages depends on the nature of the ingested cargo [18].

In order to get insights into the cellular mechanisms of heme processing and degradation, we used our previously described cellular model of EP. This model uses Ca^2+^ artificially aged RBC, phagocytosed by either quiescent or activated murine BMDM. We have previously shown that RBC processing is an early event in our cellular model of EP. Indeed, we described heme catabolism kinetics during EP and showed that this process starts as early as 1 hr post-EP [24]. Using this model and on the basis of previously established kinetics of EP, we followed subcellular localization of Nramp1, Nramp2/DMT1, Fpn, as well as HO-1, HO-2 and HRG1 during the early steps of EP. We also tested the putative recruitment of ER to the erythrophagosome using two independent methods: calnexin fluorescence labeling and glucose-6-phosphate cytochemistry.

## Results

### Recruitment of Nramp1 but not Nramp2/DMT1 at the Phagosomal Membrane Containing an Artificially Aged RBC

**Figure 1:**
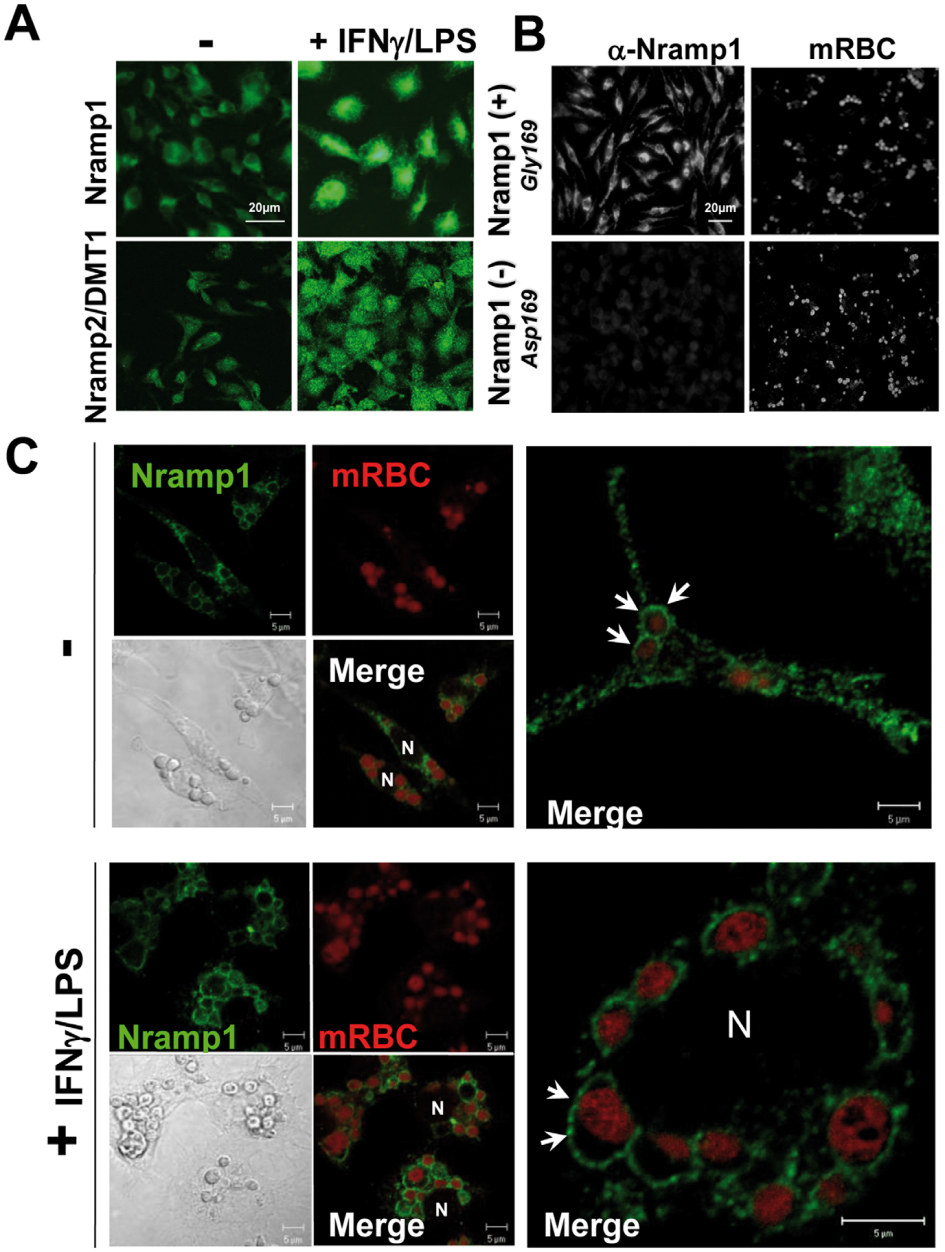
Subcellular localization of Nramp1 protein during EP. (A) IF staining of Nramp1 and Nramp2/DMT1 in untreated (−) or IFNγLPS-treated BMDM. Both Nramp1 and Nramp2/DMT1 expression are up-regulated after LPS/IFNγ treatment (B) IF of Nramp1 (left panels) was performed on quiescent Nramp1(+) or Nramp1(−) BMDM incubated with artificially-aged mRBC (1 hr). Nramp1 (+) or Nramp1 (−) BMDM present similar phagocytic activity as shown with hemoglobin autofluorescence at 520 nm (right panels) of the ingested mRBC. (C) Confocal microscopy analysis of the cellular distribution of Nramp1 (green) after EP in both quiescent (−) or LPS/IFNγ-treated BMDM. Ingested mRBC were visualized through auto-fluorescence of hemoglobin (red) or phase contrast. High magnifications (right panels) clearly show Nramp1 staining (green) at the phagosomal membrane surrounding ingested mRBC in both quiescent and activated BMDM (arrows). N: Nucleus.

Expression of the two divalent metal transporters, Nramp1 and Nramp2/DMT1 was analyzed by immunofluorescence (IF) in quiescent and activated BMDM (Figure 1). Nramp1 and Nramp2/DMT1 were detected in quiescent BMDM isolated from mice expressing the wild-type allele of Nramp1, G169 (Figures 1A and 1B left upper panels). IF staining of both Nramp1 and Nramp2/DMT1 was significantly increased in LPS/IFNγ-activated BMDM (Figure 1A, right panels). These observations were also confirmed by Western blot analysis (data not shown). As a specificity control for our anti-Nramp1 antibody and as previously described [10], the protein Nramp1 was not detected in BMDM isolated from mice expressing a mutated allele of Nramp1 (G169D, Figure 1B, left panels). Using artificially aged mRBC (see Design and Methods), we observed that the erythrophagocytic activity was similar in both Nramp1 (-) and Nramp1 (+) BMDM, as shown by ingested mRBC visualized at 520 nm (autofluorescence of hemoglobin, Figure 1B, right panels).

We next studied by confocal microscopy the subcellular distribution of Nramp1 during EP in both quiescent and LPS/IFNγ activated BMDM (Figure 1C). The presence of mRBC inside BMDM after phagocytosis was clearly observed with a phase contrast objective, or by looking at the autofluorescence of hemoglobin at 520 nm (red). Nramp1 was found to be expressed in intracellular vesicular compartments distributed throughout the cells. Upon EP, Nramp1 staining was shown to be enriched around the engulfed mRBC, delimitating the erythrophagosomal membrane (Figure 1C, arrows in upper right panel). Following LPS/IFNγtreatment, the phagocytic activity of BMDM was enhanced, as attested by higher numbers of mRBC phagocytosed per macrophage. In addition, in activated BMDM, clear enrichment of Nramp1 staining was shown at the membrane surrounding the ingested mRBC (Figure 1C, arrows in bottom right panel).

**Figure 2:**
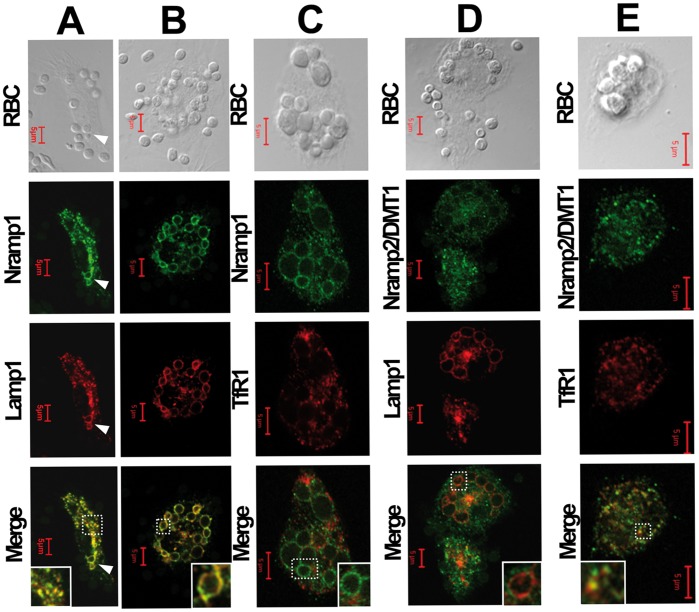
Nramp1 but not Nramp2/DMT1 is recruited to the phagosomal membrane surrounding ingested mRBC. After activation with LPS/IFNγ for 16 hrs, BMDM were processed for erythrophagocytosis assay (1 hr) and double IF labeling was performed as follows. (A and B): Nramp1 and Lamp1; (C): Nramp1 and TfR, (D): Nramp2/DMT1 and Lamp1; (E) Nramp2/DMT1 and TfR. Confocal analysis indicates that Nramp1 is present in vesicular endomembranes and is concentrated at the erythrophagosomal membrane where it colocalizes with Lamp1 (A and B) but displays a different localization than TfR (C). On the other hand Nramp2/DMT1 does not show any evidence of phagosomal membrane localization (D) but colocalizes with TfR (E) in recycling endosomes.

We then performed co-immunolabeling of both Nramp proteins, with either the lysosomal protein Lamp1 or the early recycling endosomes marker, namely the transferrin receptor 1 (TfR1) (Figure 2). Similar observations were made in both quiescent (not shown) and LPS/IFNγ-activated BMDM (Figure 2). Nramp1 strongly co-localized with Lamp1 positive vesicles, attesting for the presence of Nramp1 in lysosomes (Figure 2A). After EP, both Lamp1 and Nramp1 were strongly recruited at the erythrophagolysosomal membrane (Figure 2A and B). At a higher magnification, events of lysosomes fusion with the erythrophagosomal membrane were clearly observed (Figure 2B and DataS1A). On the other hand, Nramp1 did not show significant co-staining with TfR1, which conserved an intracellular and vesicular staining all through EP (Figure 2C). When compared to Nramp1 staining, the cellular distribution of Nramp2/DMT1 was more diffuse into the cytosol of BMDM (Figure 2D), with the presence of the transporter in small vesicular compartments. Importantly, we did not observe any co-localization with Lamp1 nor any recruitment of Nramp2/DMT1 at the phagosomal membrane during EP. This has been observed in both quiescent and activated BMDM, as well as in Nramp1 (−) BMDM (not shown). In contrast, Nramp2/DMT1 showed significant co-staining with the TfR1, indicating the strong presence of this transporter in recycling endosomes (Figure 2E). We also studied the cellular distribution of ferroportin (Fpn) after EP in BMDM (DataS2). After activation with LPS and IFNγ, the level of Fpn protein expression decreased in BMDM, as previously reported [24]. After EP, we did not detect any significant change in the vesicular staining of Fpn, with no recruitment of the iron exporter at the erythrophagosomal membrane, neither in quiescent or activated BMDM (DataS2).

### HO Proteins are not Present Inside or at the Membrane of the Erythrophagolysosome

**Figure 3:**
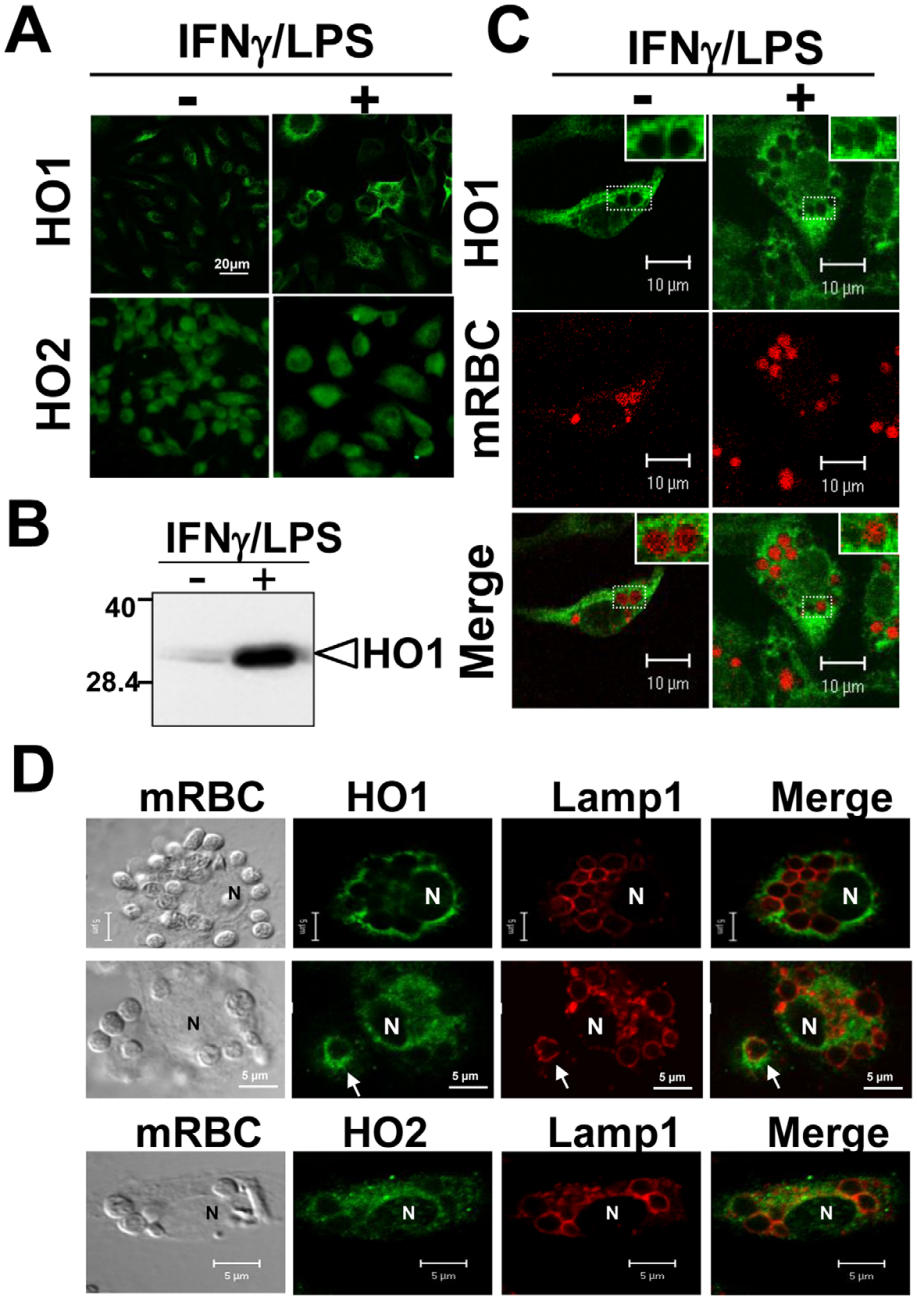
Heme oxygenase expression and localization in BMDM after activation and EP. Expression of HO-1 and HO-2 was analyzed in quiescent (−) or activated (with LPS/IFNγ) BMDM by classical fluorescence (A) or by Western blot (B; for HO-1 only). HO-1 but not HO-2 was induced after pro-inflammatory cytokines treatment. (C) Localization of HO-1 during EP (1 hr) in quiescent or activated BMDM. Mouse RBC are visualized through autofluorescence of hemoglobin (middle panels). (D) Confocal analysis of HO proteins and Lamp1 staining during EP. HO-1 and HO-2 do not colocalize with Lamp1 at the phagosomal membrane but rather display a diffuse staining around the RBC-containing phagosome.

We next evaluated the subcellular localization of HO proteins (HO-1 and HO-2) in BMDM (Figure 3). HO proteins display a diffuse intracytosolic staining in BMDM. HO-1 expression was shown to be strongly increased after LPS/IFNγ stimulation of BMDM, both by IF (Figure 3A, upper panels) and Western blot (Figure 3B) analysis. As expected, the expression of HO-2 staining remained unchanged following macrophage activation (Figure 3A, bottom panel). During EP, intracellular distribution of HO-1 did not change in control or activated BMDM, HO-1 being dispersed into the cytosol and around the erythrophagosome, with no phagosomal membrane enrichment or recruitment (Figure 3C). Confocal analysis of dual staining of HO-1 or HO-2 with Lamp1 (Figure 3D and Data S1B) indicated no co-localization of the protein studied. Our observations suggest the absence of HO proteins inside or at the membrane of mRB-containing phagolysosomes.

### ER Contribution during Phagocytosis of Senescent RBC by BMDM

**Figure 4:**
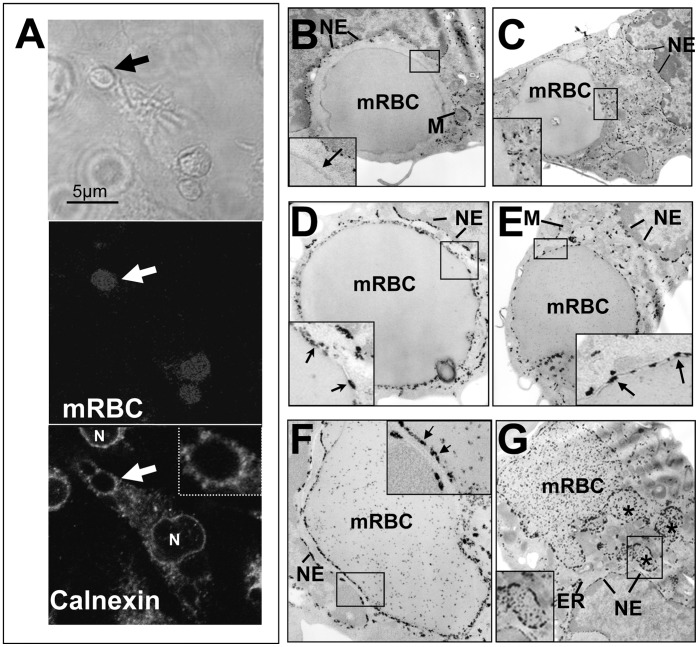
Endoplasmic reticulum and erythrophagocytosis. BMDM were incubated with mouse artificially aged erythrocytes (mRBC) for 15 (A) to 30 min (B–G) and were processed either for calnexin fluorescence labeling (A) or G6Pase activity for EM analysis (B–G). In A, engulfed erythrocytes were visualized by phase contrast (upper panel) or by hemoglobin autofluorescence at 520 nm (middle panel). Lower panel in A shows calnexin labeling around one phagosome containing a RBC (arrow and inset). The nuclear membrane (N) and the ER (intracellular staining) are specifically labeled for calnexin. (B) At the ultrastrutural level, some ingested mRBC phagosomes were negative for G6Pase. A dense area located around the membrane of the phagosome was clearly negative for any cellular structure and for G6Pase activity (see inset high magnification). This region likely corresponds to a network of actin filaments surrounding the phagosome and indicates the early nature of this phagosome. In C, some G6Pase positive patches are organized around and close to the membrane of the mRBC-containing phagosome (see inset). In most of mRBC-containing phagosomes, EM sections reveal intense G6Pase labeling that accumulates between the phagosomal membrane and the membrane of the ingested erythrocyte (D to F; see arrows in inset). Some erythrophagosomes present irregular shapes associated with appearance of intra-phagosomal electron-opaque particles that likely correspond to G6Pase precipitates that diffuse throughout the phagosome (F and G; see results and discussion). In G (see inset), asterisks indicate vesicular compartments harboring strong G6Pase activity that may illustrate fragmentation of the erythrophagosome. The nuclear envelope (NE) and endoplasmic reticulum (ER) were specifically and strongly labeled.

HO proteins have been described as enriched in the ER [25]. The ER has been shown to participate in the phagocytosis of latex beads as well as in the phagocytosis of antibody-opsonized erythrocytes in the J774 macrophage cell line [22], [23] and dendritic cells [21]. In our EP assay involving artificially aged mRBC phagocytosed by BMDM, the absence of HO-1 at the phagosomal membrane or inside the erythrophagolysosome could suggest that ER does not contribute to the formation of this specific erythrophagolysosome. To clarify this point, we studied the recruitment of two ER markers in our cellular system, and visualized the calnexin staining by IF (Figure 4A) and G6Pase activity by EM (Figure 4B–G) during EP. Calnexin is a chaperone, localized in the intra-luminal side of the ER. Due to its localization in the intracellular space of the ER, calnexin has been shown to be rapidly degraded after fusion of lysosomes with phagosomes [26]. We therefore performed short time incubation (15 min) of BMDM with mRBC and looked at fluorescence calnexin staining. As a positive control of antibody specificity, the nuclear envelope as well as the ER were specifically labeled for calnexin (Figure 4A). Calnexin was shown to be enriched at the membrane of some mRBC-containing phagosomes, likely delimitating the phagosomal membrane (see arrow in Figure 4A). The presence of calnexin at the phagosomal membrane was observed in patches around the mRBC-containing phagosomes (see enlargement of Figure 4A) suggesting the presence of calnexin in microdomains as previously observed [26].

**Figure 5:**
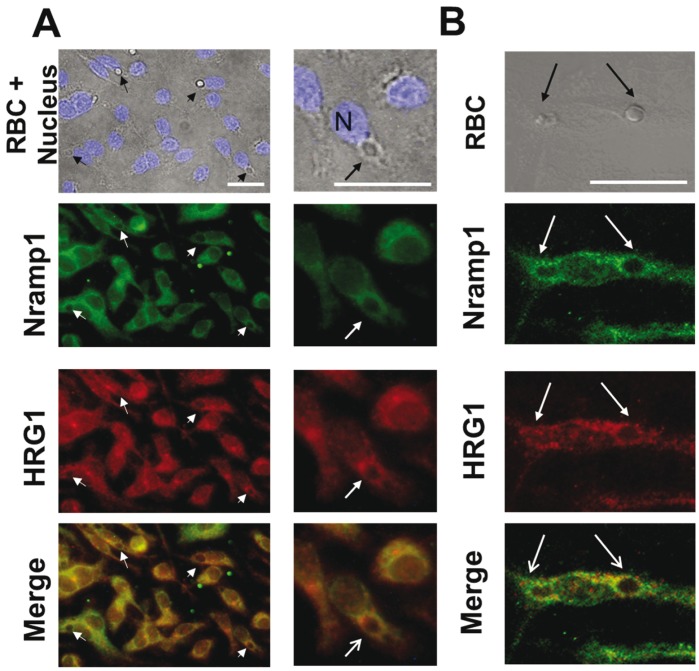
HRG1 is recruited with Nramp1 to the phagosomal membrane surrounding ingested mRBC. BMDM were processed for erythrophagocytosis assay (1 hr) and double IF labeling of HRG1 (red) and Nramp1 (green) was performed. Arrows indicate the position of erythrophagosomes. White bars in upper panels correspond to a size of 20 µm. In panel A, nuclei (N) are visualized with DAPI. Classical fluorescence (A) or confocal analysis (B) indicate that HRG1 and Nramp1 share similar localization within the cells. In addition, HRG1 and Nramp1 are both concentrated at the erythrophagosomal membrane (arrows).

Phagosomes containing artificially aged mRBC in BMDM were then studied by EM for G6Pase activity, a more stable marker of ER recruitment (Figure 4B–G) [22]. As a positive control of the specificity of the labeling, the nuclear envelope (NE) and intra-cytosolic cisternae of ER were both positive for G6Pase activity (electron dense deposits) whereas mitochondria (M) were negatively stained (Figure 4B–G). In some EM sections, few erythrophagosomes were negative for the ER marker (Figure 4B) or showed ER organization close to their membrane (Figure 4C). In most cases, we clearly observed the presence of G6Pase activity at the erythrophagosomal membrane (Figure 5D–G, arrows) as illustrated by electron dense deposits between the membrane of the phagosome and the membrane of the mRBC (see Insets in Figure 4D–F). Some erythrophagosomes (Figure 4F and G) presented a granular appearance with a less defined shape (not round). The nature of this electron-dense granular material is not clear but it may correspond to diffusion of G6Pase reaction product after mRBC membrane breakdown/degradation during the process of mRBC digestion in late phagolysosomes. In addition, in some cases, the erythrophagosome membrane showed some evidence of invaginations and erythrophagosome shrinkage (Figure 4G, see asterisks and inset). Altogether, these results indicate contribution of ER in phagosome maturation in our models using artificially aged mRBC for erythrophagocytosis.

### The Heme Transporter HRG1 Localizes with Nramp1 at the Erythophagosomal Membrane

Absence of HO recruitment to the phagosome containing a mRBC suggests that heme is not degraded into the erythrophagosome and therefore diffuses or is transported through the phagosomal membrane. To test such an hypothesis, we performed co-labeling of Nramp1 with HRG1, recently identified as a heme transporter [17] (Figure 5). Classical fluorescence and confocal analysis confirmed the presence of HRG1 with Nramp1 in cytosolic lysosomes as previously described [17]. In addition, concentration and partial colocalization of the two transporters at the erythrophagosomal membrane were observed (arrows in Figure 5).

## Discussion

### A Suitable Cellular Model of EP

The process of heme iron recycling after EP in macrophages is not completely understood. This is due in part to the use of various cellular models of EP which are described in the literature [3]. They are commonly based on macrophage cell lines (such as J774 or RAW264.7), phagocytosing various sources of heme or erythrocytes mainly as IgG opsonized RBC [1], [3], [27]. In a previous work, we characterized an original cellular model of EP that mimics the physiological pathway of ageing of RBC, followed by their recognition and engulfment by primary BMDM [24]. In this model, erythrocytes are aged by increasing their intracellular calcium content, which leads to the exhibition of several features of physiological RBC senescence also known as eryptosis [24], [28], [29]. As an example, a massive exposure of phosphatidylserine residues is observed in the outer lipid leaflet of treated RBC [24], [28] and likely participates in the recognition and the engulfment of senescent erythrocytes by macrophages expressing specific receptors [30]. Indeed, following our Ca^2+^-ageing treatment, mRBC present specific binding and phagocytosis by BMDM. As previously shown [24], LPS/IFNγ activation of macrophages increased phagocytic activity of BMDM. Interestingly, using Ca^2+^-treated RBC, the mouse macrophage cell line J774 or RAW264.7 presented a much lower phagocytic activity when compared to BMDM (Data S3). In addition, unlike the mouse macrophage cell lines J774 and RAW264.7, primary macrophages such as BMDM express high level of endogenous Nramp1, Nramp2/DMT1, HO-1 and Fpn [10], [13], [31]. These proteins are described to be involved in macrophage iron handling and recycling following EP [5], [6], [32], [33], [34], [35], [36] and their endogenous expression in macrophages appears therefore essential to study EP and heme iron recycling. In addition, LPS/IFNγtreatment leads to upregulation of Nramp and HO-1 proteins. Hence, our cellular model is appropriate to study key steps involved in heme iron recycling after EP, in both quiescent and inflammatory-activated macrophages.

### Contribution of Endomembranes to the Phagosomal Membrane

The process of phagosomes formation at the cell surface and their maturation into phagolysosomes is extremely complex and involves a highly regulated and coordinated sequence of membrane trafficking events. It is widely accepted that the invaginated plasmalemma is an important source of membrane used to form the phagosome. However, additional sources of endomembranes are required and contribute to the phagosome elaboration. Indeed, membrane fusion and fission events observed during the maturation of a phagosome involve multiple organelles and participate in the enlargement of the phagocytic cup [20]. Large scale proteomic approaches for phagosome composition analysis confirmed that in macrophages, phagosomes appear to be composed of hundreds of proteins [37] originating from various intracellular structures, including endosomes/lysosomes, post-Golgi membranes and ER [38]. Most data in this field were obtained with opsonized latex beads, therefore describing Fcγ receptor-mediated phagocytosis. In our EP model (not opsonized aged RBC), we clearly detect contribution of both lysosomes and ER to the erythrophagosome formation, with strong Lamp1 staining and G6Pase activity, respectively, detected at the membrane surrounding the phagocytosed RBC. Lamp1 is a marker of lysosomal compartment and its presence reflects the fusion of lysosomes with mRBC containing phagosome and maturation of this vesicular compartment as an erythrophagolysosome.

### Heme Transport and Heme Catabolism Following EP

Conventionally, HO proteins are described to be strongly expressed in the ER [25] and this was confirmed in our BMDM (data not shown). While ER clearly appears to participate in phagosome formation in our erythrophagocytosis model, we did not detect the presence of HO proteins inside or at the membrane of RBC-containing phagosomes. One can postulate that ER recruitment at the phagosome is a selective mechanism, involving only some parts of ER, which do not contain HO proteins. Interestingly, the ER localization of HO proteins was specified to the smooth ER and not on the rough ER membrane [39].

The presence of the heme transporter HRG1 and the absence of HO isoforms (HO-1 and HO2) at the phagosomal membrane or inside the phagosome containing a RBC strongly suggest that heme degradation occurs in the cytoplasm, rather than inside the phagolysosome. Interestingly, some studies related to the topology of HO1 in endomembranes suggest that the catalytic site of the enzyme is facing the cytosol [40], [41]. In our model, heme could be transported by HRG1 through the phagosomal membrane and subsequently be degraded into the cytosol. One cannot exclude that in addition to HRG1 transport, heme present in important amount into the phagosome during EP could also pass through the phagosomal membrane by passive diffusion to reach the cytosol. As an example, the degradation product of heme, bilirubin has been shown to diffuse through membrane although some putative transporter have been identified [42]. The hypothesis of heme degradation in the cytosol is also in agreement with our previously published results describing early transcriptional induction of expression of HO-1 and Fpn genes following 2 hours of EP, both of them being heme-regulated genes [24], [43], [44]. Indeed, such regulation of expression suggests that 1) heme can reach the nucleus rather than being catabolized in the phagosome and 2) heme extraction from hemoglobin is an early event. Moreover, we previously showed that heme catabolism begins as early as 1 hr post EP [24].

Based on our previously published kinetics of heme catabolism during EP [24], we focused our present work on the early steps of erythrophagocytosis (no more than 1 hr after RBC incubation) to characterize the biogenesis of the erythrophagosome. No difference in cellular distribution of the proteins studied was observed between quiescent and activated macrophages. However, the exact subcellular localization of HO-1 and the intracellular site of heme catabolism in later stages after EP remain important points to clarify in both quiescent and activated macrophages. Interestingly, HO proteins belong to tail-anchored proteins, which are integral membrane proteins characterized by a post-translational and highly specific mode of insertion into biological membranes [41], [45]. Such process is likely subject to regulations that could modulate the protein’s function. Indeed, *in vivo* tail-anchored proteins were shown to be inserted into a limited number of intracellular membranes, including ER, the mitochondrial outer membrane and the peroxisomal membrane [46]. Interestingly, HO-1 has recently been described to be present in various subcellular compartments, including nucleus [47], mitochondria [48], [49] and plasma membrane caveolae [50]. Moreover, following hemin treatment, HO-1 has been shown to be cut off from its C-terminal transmembrane region and then relocated to the nucleus [47] or mitochondria [49]. Despite description of different compartmentalization of HO-1 in the cell, the function of this enzyme in subcellular organelles is not completely understood. In the nucleus, HO-1 is shown to have a reduced heme metabolizing activity but strongly activates transcriptional factors important in the oxidative stress response [47]. Indeed, the degradation of heme and the liberation of heme iron rely on the activity of a multienzymatic complex formed by HO-1, biliverdin reductase (BVR) and NADPH cytochrome p450 reductase. Interestingly, in liver extract, BVR was poorly detected in microsomal fraction enriched in ER, questioning about the function of HO in such cellular compartment [49]. Recently, HO-1 has been shown to translocate to a plasma membrane compartment in endothelial cells upon LPS stimulation [50]. Studying the subcellular trafficking of the HO-1 multiple enzyme complexes during EP in both quiescent and activated macrophages could therefore help to define the cellular site(s) of heme catabolism and the subsequent process of heme iron recycling.

### Recruitment of Nramp Transporters

Nramp2/DMT1, the first characterized mammalian iron transporter [12], [51] is often described as a potent candidate for transporting iron from the phagosome to the cytosol. However in our model Nramp2/DMT1was not recruited at the erythrophagosomal membrane, even in the absence of Nramp1 expression (BMDM derived from balb/c mice), suggesting absence of redundancy of function of these two transporters at that site. The lack of recruitment of this iron transporter at the erythrophagosomal membrane is compatible with the hypothesis of heme catabolism occurring outside the phagosome as described before. Nramp2/DMT1 was previously shown primarily expressed in recycling endosomes in macrophages, where it likely plays a role in iron acquisition through the transferrin-transferrin receptor cycle [13]. Indeed, in the present work, Nramp2/DMT1 exhibited a strong signal in early endosomes where it colocalized with TfR. However, other studies indicate that Nramp2/DMT1 rather localizes in late endosomal or lysosomal compartments [52], [53]. Nramp2/DMT1 was also found associated with membranes of phagosomes in macrophages and Sertoli cells [52]. Differences in experimental design including between phagocytic cells [primary BMDM (this study) versus Raw macrophage cell line], ingested particles [senescent RBC (our study) versus latex beads or IgG-coated RBC] and expression system [endogenous (our study) versus overexpression of a cMyc-Tagged version of Nramp2] could likely explain this discrepancy.

Contrasting with Nramp2/DMT1, Nramp1 colocalized strongly with Lamp1 at the erythrophagosomal membrane confirming the previously described localization of Nramp1 in lysosomes. Various experimental evidences favor the function of Nramp1 at the phagosomal membrane as a pH-dependent divalent metal efflux pump (including Fe^2+^) [54]. However according to our observations at the erythrophagosomal membrane (absence of HO and presence of HRG1) it is unlikely that Nramp1 is involved in heme iron transport at that site. As it can transport various divalent metals such as Zn^2+^ and Mn^2+^, Nramp1 could be involved in the recycling of such ions from the phagosome following phagocytosis of RBC, these ions being highly concentrated in erythrocytes [55]. Interestingly, Jabado and al. previously described an Nramp1-mediated pH-dependent transport of Mn^2+^ from the phagosomal space to the cytosol through the phagosomal membrane [56]. In addition, Nramp1 was found to transport Mn^2+^ more efficiently than Fe^2+^, suggesting that Mn^2+^ could be the natural substrate for Nramp1 at the phagosomal membrane [11]. On the other hand, although several observations *in vitro* and *in vivo* suggest a role of Nramp1 in macrophage iron handling and recycling [32], [33], [34], [35], [36], [57], [58], the exact mechanism involved remains unclear. Thus, it has been proposed that the Nramp1 dependent process implies a direct lysosomal secretory pathway rather than a direct efflux of iron via the cytoplasm [32]. Indirect effect of Nramp1 expression and divalent metal ion transport has also been described in the survival of intracellular pathogens, the presence of Nramp1 affecting phagosomal maturation of *Salmonella*-containing phagosomes [59], [60]. The biochemical function of Nramp1 at the erythrophagosomal membrane could then rely on the maturation of the erythrophagolysosome and the degradation of RBC content, including hemoglobin. Experiments are still needed to clarify such roles of Nramp1 in EP.

**Figure 6:**
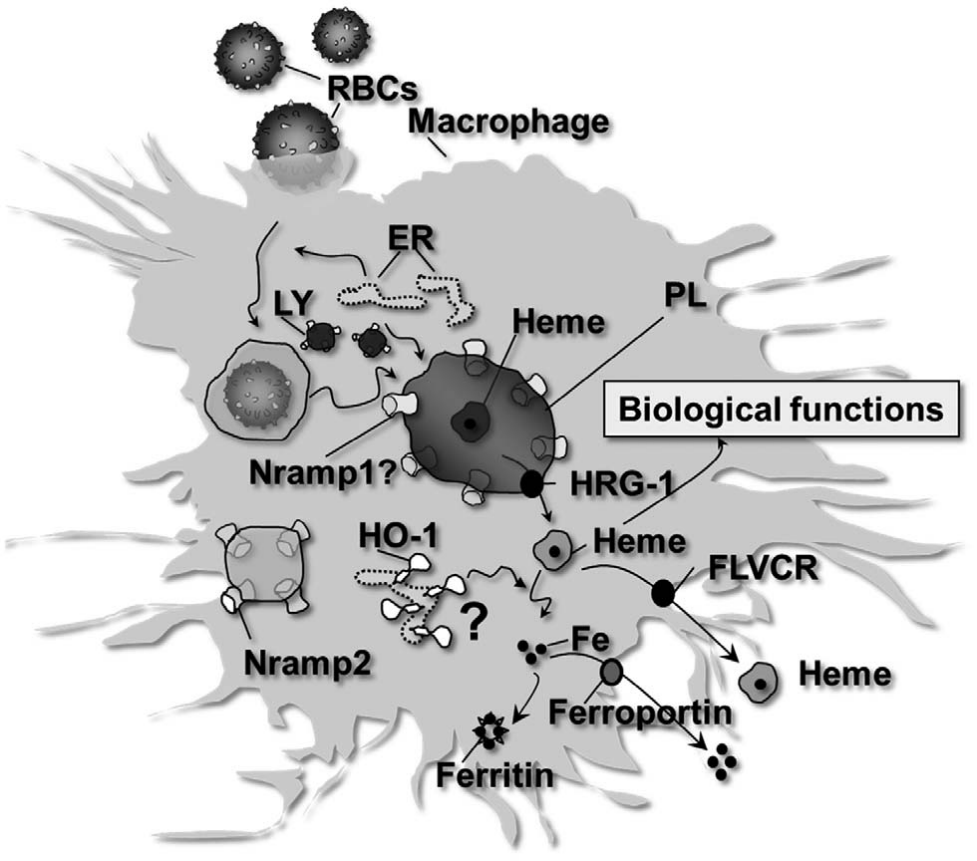
Proposed model of heme iron recycling following EP of artificially-aged RBC. Senescent RBC are specifically recognized and engulfed by BMDM inside an erythrophagosomal compartment. ER and lysosomes (LY) fuse with this compartment to form a phagolysosome (PL). During this process, Nramp1 is recruited at the phagosomal membrane. The exact role of Nramp1, strongly present at that site, still remains to be elucidated. Nramp2, primarily located in recycling endosomes, does not seem to be involved in this process. After hemoglobin degradation, heme is transported through the phagosomal membrane via HRG-1. Once in the cytosol, heme can exert its biological function and is then degraded by HO-1. Further investigations are necessary to clarify the subcellular site of heme catabolism by HO-1. Additionally, heme is transported outside the cell via FLVCR. Freed heme iron is then recycled to the circulation via Fpn or is stored in the form of cytosolic ferritin.

In conclusion, our observations delineate the early steps of EP and heme catabolism (see Figure 6). They argue against heme catabolism inside the phagosome during EP, but rather favor a transport of heme into the cytosol. Our results, together with previous ones [24], [43] therefore propose the existence of a labile heme pool (namely heme that is not a component of hemoproteins) which can exert its biological functions. Indeed, after EP, heme was shown to act as a transcriptional regulator of genes involved in iron metabolism, such as HO-1 itself or Fpn [43], [44]. Heme transport within the cell needs to be better characterized but likely involves chaperones (heme binding protein) [15]. Another important challenge will be to define the subcellular site(s) of heme catabolism by the multienzymatic complex associated with HO-1 in both quiescent and activated erythrophagocytosing macrophages.

## Materials and Methods

### Ethics Statement

Animals (mice) were cared for in accordance with criteria outlined in the European Convention for the Protection of Laboratory Animals. Animal studies received approval from the review board of Hôpital Bichat.

### L-Cell-Conditioned Medium (LCCM) Preparation

L929 cells (Cell line ATCC;NCTC clone 929) were cultured in RPMI-glutamax supplemented with 10% heat-inactivated fetal calf serum (FCS, Gibco), 2mM L-glutamine, 50 U/mL penicillin, and 50 mg/mL streptomycin. For LCCM production, cells were plated at a density of 2.5×10^4^ cells/ml in 75 or 150 Ti flasks and grown for 8 days until confluence. Cell culture supernatant was then filtered through 0.22 µm filters, aliquoted and stored at –20°C until further usage. LCCM is a source of CSF-1.

### Bone Marrow Derived Macrophage (BMDM) Culture

Bone marrow cells were isolated as previously described [24] from femurs of either C57B6/DBA2/129sv (mixed background), DBA2, 129sv (Nramp1G169, wild type) or balb/c (CharlesRiver lab) mice carrying a null mutation at Nramp1 gene (Nramp1^−/−^). Briefly, mice were sacrificed, femur were removed from hind legs and bone marrow cells were gently flushed with HBSS to be isolated from femurs. Cells were seeded at a density of 2,5×10^5^ cells/ml in Petri dishes for protein analysis and electron microscopy (EM). For immunofluorescence (IF) studies, cells were seeded at a density of 1,25×10^5^ cells/ml onto circle glass coverslips (10 mm diameter) in 24-well tissue culture plates (Falcon). The culture medium was RPMI-glutamax (Gibco) supplemented with 10% heat-inactivated FCS (Gibco, low endotoxin content), 10% LCCM, 2mM L-glutamine, 50 U/mL penicillin, and 50 mg/mL streptomycin. At 4 days after seeding, the adherent cells were rinsed twice with Hanks balanced salt solution (HBSS) containing 10 mM N-2-hydroxyethylpiperazine ethanesulfonic acid (HEPES). Medium was then renewed each day until day 7. For cellular activation, BMDM were treated overnight with interferon gamma (IFNγ, 50 U/ml, BD Biosciences Pharmigen) and lipopolysaccharide (LPS, 10 ng/ml, Sigma).

### RBC Treatments

Mice were anesthetized by isoflurane inhalation and blood was collected on ethylenediaminetetraacetic acid (EDTA) by puncture of the orbital sinus. After 3 washes with phosphate buffer saline (PBS), mouse red blood cells (mRBC) were resuspended in Hepes buffer (10 mM Hepes, 140 mM NaCl, BSA 0.1%, pH 7.4). For *in vitro* RBC ageing, cells (1×10^8^ cells/ml in Hepes buffer) were incubated overnight at 30°C with calcium (2,5 mM) and the ionophore A23187 (0,5 µM; from Streptomyces chartreuses, Calbiochem, La Jolla, CA, USA). Treated RBC were then centrifuged (1000 rpm 5 min), washed twice with PBS and resuspended in Hepes buffer before use for phagocytosis assay.

### Phagocytosis Assay

Macrophages were incubated with calcium-treated mRBC (3×10^7^ cells/ml; 1 ml/well in 24-well plate or 5 ml/in Petri dishes 3.5 cm) for the indicated times at 37°C in a 5% CO2 incubator and then washed twice with HBSS. In general (except for pictures in Figure 3A, 3B, 3D & 4D), cells were then incubated for 5 min in hypotonic solution (140 mM NH_4_Cl, 17 mM Tris-HCl pH 7.6) to lyse non-ingested RBC. Cells were then washed again twice with PBS and processed for immunostaining. For EM analysis, cells were washed twice with HBSS after EP and then processed for G6Pase activity.

### Antibodies

Polyclonal rabbit antisera against Nramp1, DMT1/Nramp2, and Fpn were produced and affinity-purified as previously described [13], [31], [51]. The rat monoclonal anti-Lamp1 (Lysosomal associated membrane protein1, clone 1D4B) antibody produced by J. Thomas August was obtained from the Developmental Studies Hybridoma bank (DSHB), developed under the auspices of the NICHD and maintained by the University of Iowa, Department of Biological Sciences, Iowa City, IA 522242. The rat anti-Transferrin receptor (TfR, CD71) was from Biosource International (Camarillo, CA). Both rabbit polyclonal anti-human heme oxygenase 1 (HO-1) or anti-rat HO-2 were from Stressgen Biotechnologies Corp (Victoria, BC Canada). The rabbit polyclonal antibody raised against the cytoplasmic domain of calnexin was produced as previously described [22]. The goat anti-HRG1 [SLC48A1 (C-15)] was purchased from Santa Cruz. Goat anti-rat Ig-Cy3 (GAR-Cy3) and goat anti-rabbit IgG–Alexa 488 (GAR-Alexa488) secondary antibodies were respectively from ImmunoResearch Lab and MolecularProbes.

### IF and Confocal Analysis

After cell culture, BMDM were fixed in 100% methanol at -20°C for 15 min. Cells were then washed twice with PBS and permeabilized with Triton X100 (0.1% in PBS) for 10 min. After two washes in PBS, cells were incubated in a blocking solution (1% BSA and 10% heat-inactivated goat serum in PBS) for 45 min at room temperature (RT). Incubation with primary antibodies was performed in a humid chamber at RT for 1 hr using the following dilutions in blocking solution: anti-Nramp1(1/200), anti-Nramp2/DMT1 (1/50), anti-Lamp1 (1/500), anti-HO-1 (1/1000), anti-HO-2 (1/500), anti-TfR (1/100) and anti-calnexin (1/200), anti-HRG1 (1/25). After three washes with PBS/0.5% BSA, cells were incubated for 1 hr at RT with secondary antibodies (GAR-Alexa 488 and/or GAR-Cy3) diluted at 1/200 in blocking solution. Slide coverslips were then washed 3 times in PBS/0.5% BSA, once in PBS and then mounted with antifade mounting agent (Prolong Antifade kit P-7481 MolecularProbes). IF was analyzed either with a Nikon microscope using the 40X or 60X oil immersion objectives or a Zeiss laser confocal microscope with a 63X objective.

### Western-blot Analysis

Crude membrane proteins extracted from cells (as previously described [61]) were solubilized in 1X Laemmli buffer and incubated for 30 min at RT prior to SDS-PAGE electrophoresis and electro-transfer on a polyvinylidene fluoride (PVDF) membrane. To control loading and transfer, membranes were stained with Ponceau red after transfer, and subsequently preincubated with blocking solution [7% skim milk in TBST (0.15% Tween20, in Tris buffered saline)] before detection. Membranes were then incubated overnight at 4°C with the following primary antibodies: Nramp1 (1/2000), Nramp2/DMT1 (1/200), HO-1 (1/4000) and Fpn (1/200). After washing with TBS-T, blots were incubated with donkey peroxidase-labeled anti-rabbit IgG (1/3000, Nordic Immunologic, Tebubio, le Perray en Yvelines, France) for 1 hr at RT and revealed by Enhanced ChemiLuminescence (ECL Pierce).

### Glucose-6-phosphate Labeling and EM

The procedure used for the cytochemistry of G6Pase was previously described by Griffiths et al (1983) [62]. Briefly, cultured cells in Petri dishes (3,5cm) were fixed after EP in 0,5% glutaraldehyde in 100 mM PIPES, pH 7.0, containing 5% (wt/vol) sucrose for 30 min at 4°C, washed in the same buffer containing 10% (wt/vol) sucrose and then briefly washed in Tris-maleate 0,08 M pH 6.5. To reveal G6Pase activity, cells were incubated for 2 hrs at RT in a solution (1 ml per Petri dish) obtained by dissolving 0,19 g of glucose-6-phosphate in 10 ml of Tris-maleate 0,08 M pH 6.5 and slowly adding 80 µl of a 12%(wt/vol) solution of lead nitrate. After incubation, cells were rinsed in Tris-maleate 0,08 M pH 6.5, in 0,1 M cacodylate buffer pH 7,2–7,4 and then fixed in 2,5% glutaraldehyde in 0,1 M cacodylate buffer for overnight at 4°C. Osmication was performed using 1% aqueous osmium tetroxide (OsO4) and 1,5% aqueous potassium ferrocyanide for 1 hr at 4°C. Cells were then washed with 0,1 M cacodylate buffer and then with 0,05 M NaOH-maleate buffer pH 6.0 followed by 1 hr incubation at 4°C in 2% uranyl acetate in 0,125 M maleate buffer. Cells were then rinsed in 0,05 M NaOH-maleate buffer pH 6.0, dehydrated in ethanol and embedded in EPON. Utrathin sections were stained first in 3% uranyl acetate, counterstained with Reynold’s lead citrate and visualized on a Zeiss CE902 transmission electron microscope.

## Supporting Information

Data S1 **High magnification of Nramp1, HO-1, HO2 and lamp1 staining around a phagolysosome.** (A) Nramp1 and Lamp1 are strongly enriched at the phagosomal membrane containing a RBC. White arrows heads indicate fusion events of lysosomes at the erythrophagosomal membrane. (B) Confocal analysis indicates no colocalisation of HO-1 or HO2 with Lamp1 at the membrane of the erythrophagolysosome.
(TIF)

Data S2 **Ferroportin expression and localization in BMDM after activation and erythrophagocytosis.** Expression of Fpn was analyzed in quiescent (−) or activated (with LPS/INFg) BMDM by western-blot (A) or classical fluorescence (B). Ferroportin expression was strongly reduced after pro-inflammatory cytokines treatment as previously described (Delaby C. et al, ECR 2005). (B) Localization of Fpn during EP (1 hour) in quiescent or activated BMDM. RBC are visualized through auto-fluorescence of hemoglobin (middle panels). Ferroportin display a vesicular staining into the cytosol and some concentration at the cell surface of BMDM, as previously observed (Delaby C. et al, Blood 2005). During EP, Fpn did not show any sign of recruitment at the phagosomal membrane.
(TIF)

Data S3 **Determination of intracellular heme content after EP in different macrophages populations**. Following incubation of macrophages with Ca^2+^ treated RBC (1 hour), intracellular heme content was determined according to the method of Motterlini et al (*Am J Physiol, 1995*). Briefly, cells were washed with PBS, counted, centrifuged and the pellet was then solubilized by adding 500 µl of concentrated formic acid. The heme concentration of the formic acid solution was determined spectrophotometrically at 400 nm and normalized to the number of cells. Primary BMDM exhibited a strong EP activity with a 20 times increase of intracellular heme whereas J774 and Raw only showed in the same experimental condition a low increased of heme concentration.
(TIF)
