# Correction: Subcellular Localization of Iron and Heme Metabolism Related Proteins at Early Stages of Erythrophagocytosis

**DOI:** 10.1371/annotation/d301d160-bbe0-4d48-847f-b8a57aa853c0

**Published:** 2012-12-07

**Authors:** Constance Delaby, Christiane Rondeau, Cécile Pouzet, Alexandra Willemetz, Nathalie Pilard, Michel Desjardins, François Canonne-Hergaux

There was an error in affiliation 1 for authors Constance Delaby and François Canonne-Hergaux. Affiliation 1 should be: INSERM, U773, Centre de Recherche Bichat Beaujon CRB3, Université Paris Diderot, site Bichat, Paris, France, 

