# Supplementary figures and images for: Subcellular Localization of Iron and Heme Metabolism Related Proteins at Early Stages of Erythrophagocytosis
Source: PLoS One. 2012 Jul 30;7(7):e42199. doi: 10.1371/journal.pone.0042199 (PMC3408460; doi:10.1371/journal.pone.0042199)

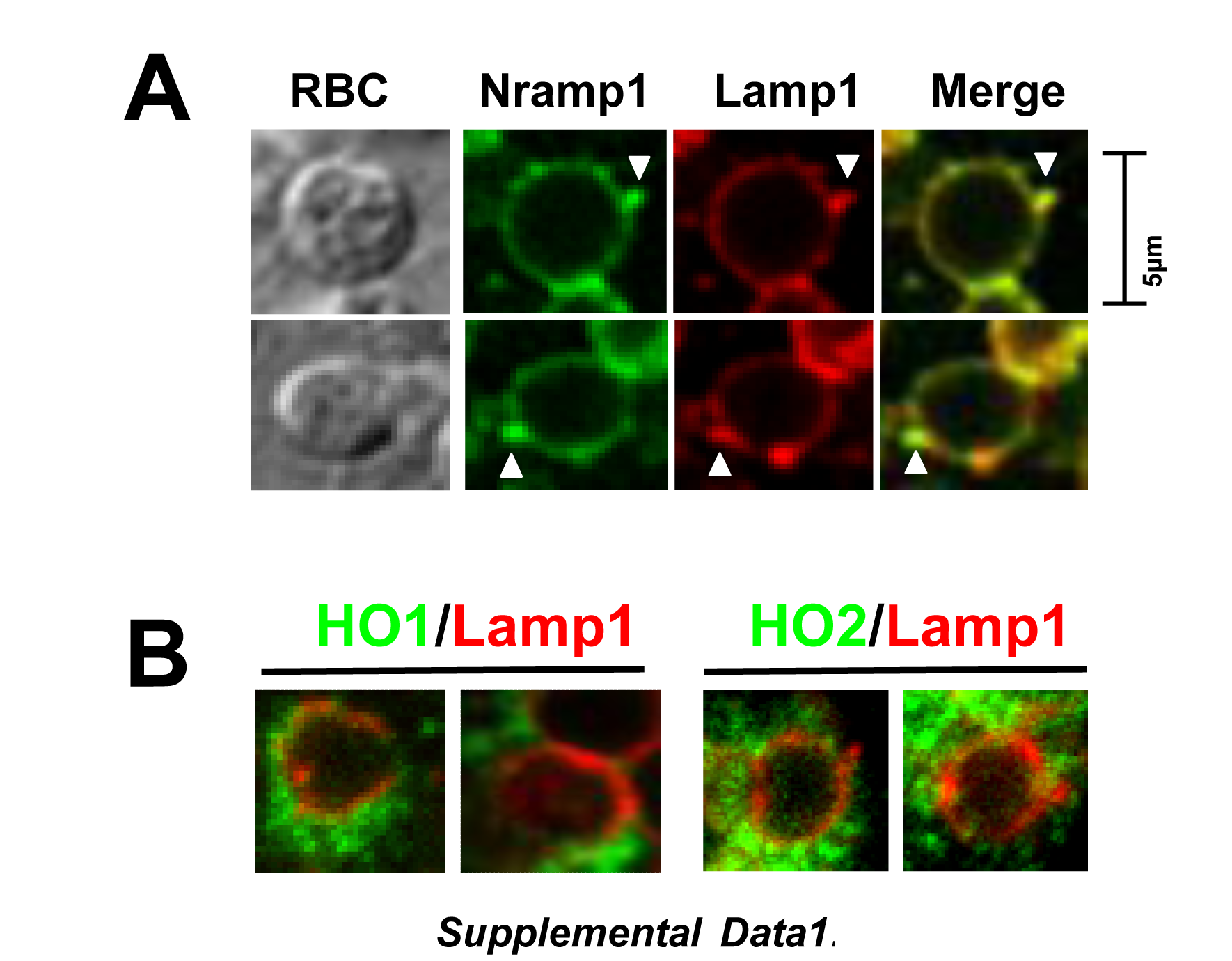

Supplement: Data S1 — High magnification of Nramp1, HO-1, HO2 and lamp1 staining around a phagolysosome. (A) Nramp1 and Lamp1 are strongly enriched at the phagosomal membrane containing a RBC. White arrows heads indicate fusion events of lysosomes at the erythrophagosomal membrane. (B) Confocal analysis indicates no colocalisation of HO-1 or HO2 with Lamp1 at the membrane of the erythrophagolysosome. (TIF) [file pone.0042199.s001.tif]

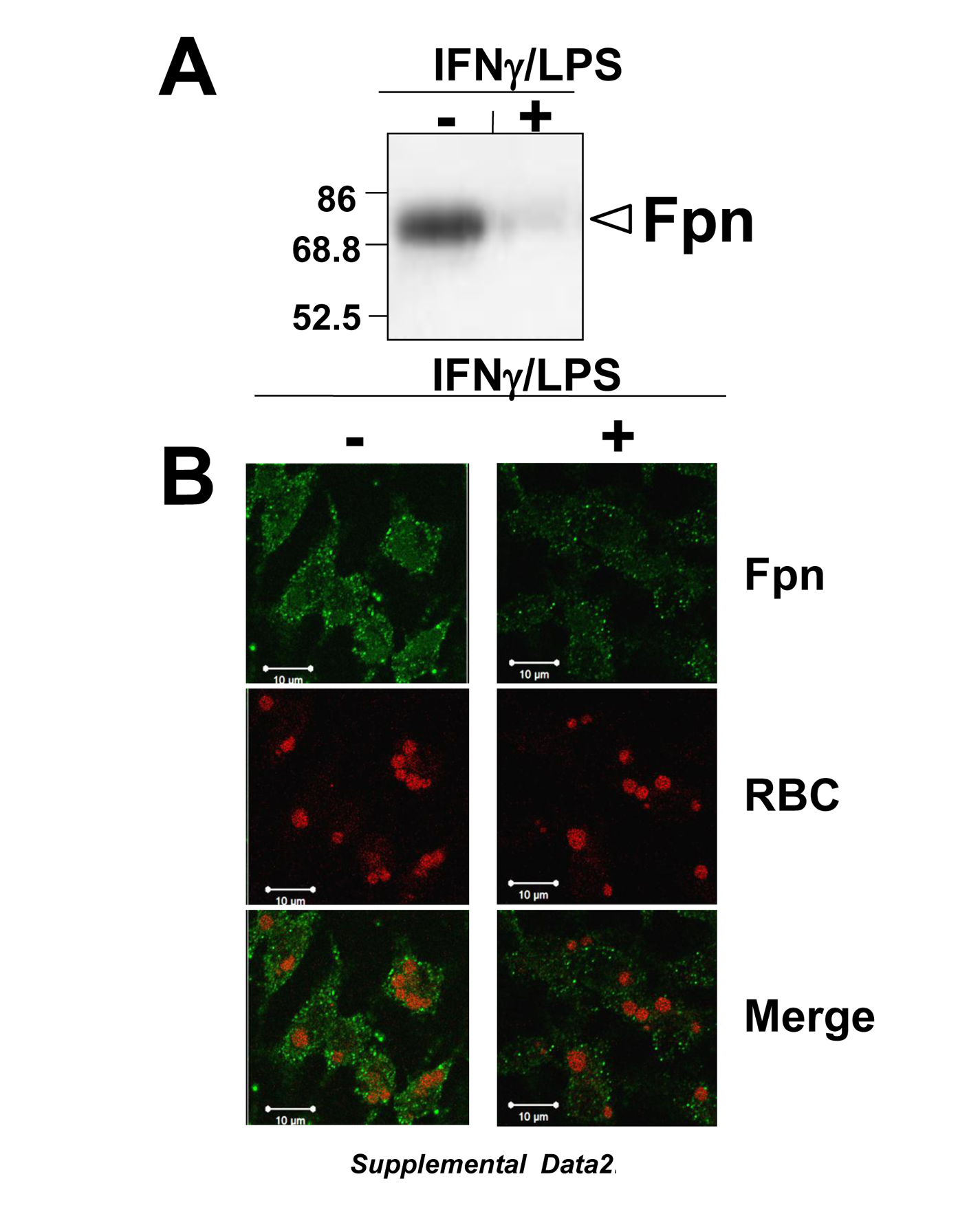

Supplement: Data S2 — Ferroportin expression and localization in BMDM after activation and erythrophagocytosis. Expression of Fpn was analyzed in quiescent (−) or activated (with LPS/INFg) BMDM by western-blot (A) or classical fluorescence (B). Ferroportin expression was strongly reduced after pro-inflammatory cytokines treatment as previously described (Delaby C. et al, ECR 2005). (B) Localization of Fpn during EP (1 hour) in quiescent or activated BMDM. RBC are visualized through auto-fluorescence of hemoglobin (middle panels). Ferroportin display a vesicular staining into the cytosol and some concentration at the cell surface of BMDM, as previously observed (Delaby C. et al, Blood 2005). During EP, Fpn did not show any sign of recruitment at the phagosomal membrane. (TIF) [file pone.0042199.s002.tif]

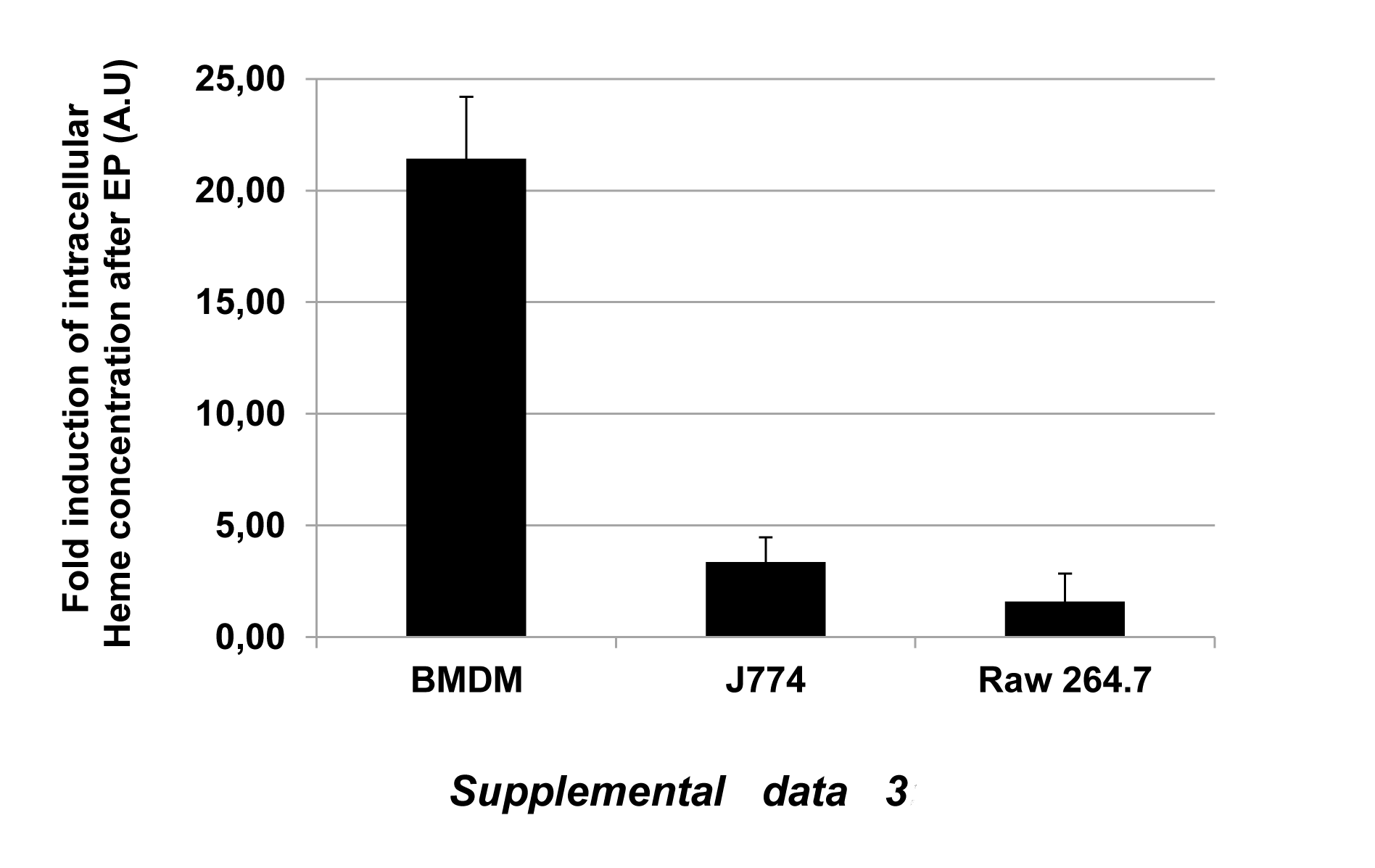

Supplement: Data S3 — Determination of intracellular heme content after EP in different macrophages populations. Following incubation of macrophages with Ca2+ treated RBC (1 hour), intracellular heme content was determined according to the method of Motterlini et al (Am J Physiol, 1995). Briefly, cells were washed with PBS, counted, centrifuged and the pellet was then solubilized by adding 500 µl of concentrated formic acid. The heme concentration of the formic acid solution was determined spectrophotometrically at 400 nm and normalized to the number of cells. Primary BMDM exhibited a strong EP activity with a 20 times increase of intracellular heme whereas J774 and Raw only showed in the same experimental condition a low increased of heme concentration. (TIF) [file pone.0042199.s003.tif]
